# The Expression of Selected Cytokine Genes in the Livers of Young Castrated Bucks after Supplementation with a Mixture of Dry *Curcuma longa* and *Rosmarinus officinalis* Extracts

**DOI:** 10.3390/ani13223489

**Published:** 2023-11-12

**Authors:** Daria Maria Urbańska, Marek Pawlik, Agnieszka Korwin-Kossakowska, Karolina Rutkowska, Ewelina Kawecka-Grochocka, Michał Czopowicz, Marcin Mickiewicz, Jarosław Kaba, Emilia Bagnicka

**Affiliations:** 1 Department of Biotechnology and Nutrigenomics, Institute of Genetics and Animal Biotechnology Polish Academy of Sciences, ul. Postepu 36A, 05-552 Jastrzebiec, Poland; 2 Department of Neurotoxicology, Mossakowski Medical Research Institute Polish Academy of Sciences, ul. Pawińskiego 5, 02-106 Warsaw, Poland; pawlik.marek@gmail.com; 3 Department of Medical Genetics, Medical University of Warsaw, Pawińskiego 3c, 02-106 Warsaw, Poland; 4 Division of Veterinary Epidemiology and Economics, Institute of Veterinary Medicine, Warsaw University of Life Sciences-SGGW, Nowoursynowska 159c, 02-776 Warsaw, Poland; michal_czopowicz@sggw.edu.pl (M.C.); marcin_mickiewicz@sggw.edu.pl (M.M.); jaroslaw_kaba@sggw.edu.pl (J.K.)

**Keywords:** castrated buck, cytokines, gene expression, liver, turmeric-rosemary mixture

## Abstract

**Simple Summary:**

Turmeric (*Curcuma longa*) and rosemary (*Rosmarinus officinalis*) are broadly used in many cuisines as spices/herbs and, containing active components, they contribute to reducing inflammation. As goats can serve as the animal model of some human conditions, our findings can broaden our knowledge of human metabolism and the impact of supplements on human health. The study aims to determine the effect of supplementation with a mixture of dried turmeric and rosemary extracts on the cytokine gene expressions in the livers of twenty eight-month-old castrated goat bucks. Their mean live weight at the beginning of experiment was approx. 30 kg. In the treated group a dose of 1.6 g/day/buck was administered orally for 124 days. After slaughter, liver fragments were collected. Total RNA was then isolated and RT-qPCR was performed. Treatment resulted in reduced *IL-12* expression and higher *IL-18* expression which may indicate a hypersensitivity reaction caused by an excessive supplement dose. Increase in the *IFN-γ* expression was also noted which may indicate that the supplement has pro-inflammatory effect. The observation that a small dose of mixture can induce an allergic reaction in young bucks suggests that high levels of curcumin and/or turmeric extract may exert some impact on the immunological system function of humans.

**Abstract:**

The study aims to determine the effect of supplementation with a mixture of *Curcuma longa* and *Rosmarinus officinalis* extracts (896:19 ratio) on the expression of 15 cytokine genes in the livers of 20 castrated goat bucks. Two equal groups were created: treated and control groups. The treated group was provided a mixture (1.6 g/day/buck) for 124 days. Liver tissue samples were collected after slaughter. The gene expression was analyzed using RT-qPCR with two reference genes. Variance analysis was conducted using a model with the group fixed effect. *IL-2* and *IL-8* expression was below the detection level. No differences were found for *IL-1α*, *IL-1β*, *IL-4*, *IL-6*, *IL-10*, *IL-16*, *IFN-α*, *IFN-β*, *TNF-α*, and *CCL4* expressions, suggesting that supplementation does not activate cytokine production in the healthy hepatocytes. The treated group demonstrated lower *IL-12* expression (*p* < 0.05) and a tendency for higher *IL-18* and *INF-γ* (0.05 *< p* < 0.10) expressions, which may indicate a hypersensitivity resulting from excessive supplement dose. The increased *IFN-γ* expression could be caused by the increased *IL-18* expression. If a small dose of extract can induce an allergic reaction in young goat bucks, it is also possible that humans may be susceptible to an overdose of curcumin and/or turmeric extracts.

## 1. Introduction

The basic requirement for the development of livestock farming and the health of the animals is to ensure their welfare. Recent years have seen growing demands from consumers regarding animal welfare to both ethical issues, and the fact that valuable and healthy products can be only obtained from healthy animals [1]. Although goats have a higher level of resistance to diseases than other ruminant species [2], low animal immunity remains a problem in intensive farming. For this purpose, it has become increasingly common to use nutraceuticals in disease prevention [3].

Nutraceuticals are food or feed that contains a bioactive component and can be regarded as a combination of nutrition and pharmaceuticals [4,5]. Nutraceuticals can be used to improve animal health, but have to be administered at higher doses than can be found in regular feeds [4]. Thanks to their beneficial effects on animal health, new nutraceuticals are increasingly being sought in plant species thought to have potential benefits for small ruminants [5]. Many substances supplied in the diet have a significant impact on the intensity, course, and relief of inflammation. Two such substances known to strongly contribute to reducing inflammation are turmeric (*Curcuma longa*) and rosemary (*Rosmarinus officinalis*). Hence, these are promising candidates for use in the diet of ruminants [4,6]. Curcumin is the main polyphenol in turmeric. It is a highly potent antimicrobial agent with antioxidant, anti-inflammatory, anticancer, and antidiabetic properties, while rosemary contains a number of natural polyphenols, such as carnosol, carnosic acid, rosmarinic acid, rosmanol, and caffeic acid. These compounds also demonstrate antioxidant, antiviral, and anticancer properties [7]. Turmeric reduces the production of pro-inflammatory cytokines (including interleukins and chemokines) such as TNF-α, IFN-α and IL-6, as well as IL-8, IL-17, chemokine (C-X-C motif) ligand 1 (CXCL1), CXCL5, and CXCL12 [8]. It also increases the expression of anti-inflammatory cytokines such as IL-10 and IL-11 [9].

Supplementation with rosemary or turmeric alone has been found to influence the profile of cytokine expression. Therefore, it is reasonable to assume that they pair may exert a synergistic effect on the immune system when used in combination. This may be confirmed by performing a comprehensive analysis of the cytokine expression profile. The results will provide an insight into the complex nature of the interactions between cytokines in response to the herbal mixture and illustrate how the expression profile of one influences those of the others.

The mixture consists of dried *C. longa* and *R. officinalis* extract in a ratio of 896:19. It is distributed as a commercial product by Trouw Nutrition, a Nutreco company (Amersfoort, The Netherlands). The ratio of *C. longa* to *R. officinalis* was established by the manufacturer based on previous experiences and studies on dairy cows (https://www.trouwnutrition.com/en/programme-lister/ao-mix-170937/; accessed on 27 September 2023). The producer indicates that the polyphenols in the mixture act as antioxidants, support performance and the immune system, and prevent lipid oxidation, and hence can partially replace vitamin E. A previous experiment conducted on adult dairy goats found the supplement to demonstrate no impact on milk yield or its components, nor on the expression of antimicrobial peptide genes (goat defensin 1 and 2) [10]. Therefore, the present study examines its influence on younger animals. Previous studies have found the mixture to have a beneficial impact on the growth performance of castrated bucks; it positively influenced body mass, daily gain, cold carcass weight, and slaughter performance. Moreover, the extracts had no apparent effect on the lipid metabolism of the castrated bucks or the culinary quality of their meat [11].

An animal is protected against disease by the proper functioning of its immune system, of which one of the main weapons is a large group of proteins from the cytokine family. They are produced by almost all cells of the organism and present a wide spectrum of activity. Pro-inflammatory cytokines induce and maintain inflammation, with the aim of defeating infections, repairing damage, and eradicating pathogens. Amongst the various mediators of the inflammatory reaction, cytokines play a key role. Anti-inflammatory cytokines are also present in the inflammation area but reveal their action at other times [12,13,14]. 

The level of pro-inflammatory cytokines serves as an indicator of the degree of activation of the immune system. As these cytokines play a vital role in the immune defense system, and the polyphenols contained in *C. longa* and *R. officinalis* are known to influence the expression of the cytokine genes, our hypothesis was that the turmeric-rosemary dry mixture will also influence the immune system of young, castrated bucks.

As goats can also serve as animal models for humans, our findings can broaden the knowledge of human metabolism, particularly as both turmeric and rosemary are considered to have a number of health benefits, including protecting against paracetamol overdoses [15,16]. Furthermore, curcumin is also used as a food coloring agent. However, some studies indicate that curcumin has a harmful influence on the liver, and can induce injury due to inflammation, oxidative stress, and metabolic disorders caused by overdose [17].

Thus, the aim of our study was to determine the effect of supplementation with a mixture of dried *C. longa* and *R. officinalis* extracts on the expression of selected cytokine genes in the livers of young, castrated goat bucks of the Polish White Improved (PWI) breed. It also examined whether the recommended dose is suitable for animals of lower weights than adult goats and which are not lactating.

## 2. Materials and Methods

### 2.1. Animal Material

Twenty eight-month-old PWI bucks, castrated at the age of three months, were used in the study. The animals were maintained in the Experimental Farm of the Institute of Genetics and Animal Biotechnology Polish Academy of Sciences, located in Central Poland. The PWI is a single-purpose breed with strict seasonality of reproduction, i.e., the end of August to the beginning of December. As such, all bucks were born between the end of February and beginning of March. All bucks were free from small ruminant lentivirus (SRLV) infection, as confirmed by serological tests at six months of age and again just before the beginning of the study, as part of an ongoing project concerning SRLV eradication from this herd [18]. The mean live weight at the beginning of the experiment was 28.80 kg (±4.93 kg). 

The bucks were fed according to a system developed by the Institut National de la Recherche Agronomique (INRA) of France and adapted to the nutritional value of feed used in Poland [19]. They were maintained together in the same pen and fed ad libitum with good quality hay, oat grains, and wheat bran, with the addition of a vitamin and mineral mixture routinely used in the herd (Vitamix C, Polmass, Bydgoszcz, Poland). The animals received water ad libitum. All animals were provided free access to mangers and waterers, without individuals lower in the hierarchy being driven away.

The bucks were divided into two groups: a treated group (*N* = 10) and a control group (*N* = 10). All were maintained in the same environmental conditions and on the same basal diet. In the treated group, the diet was supplemented with a mixture of dried *C. longa* and *R. officinalis* extracts of ratio 896:19 (Selko^®^ AOmix, Trouw Nutrition Polska sp. z o.o., Grodzisk Mazowiecki, Poland) for 124 days from the middle of September to the middle of January. A dose of 1.6 g/day/buck was administered orally to each buck as starch capsules before morning feedings. Although this dose was recommended by the producers, it was originally established for dairy lactating goats basing on previous studies on dairy cows. 

**Figure 1:**
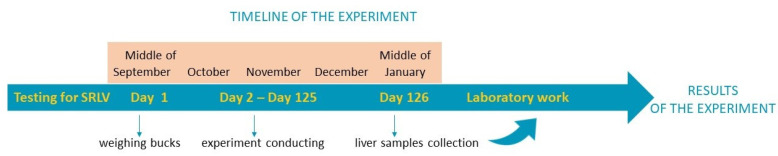
Timeline of the experiment.

Just after slaughter in a certified slaughterhouse, liver fragments (1.5 cm by 1.5 cm by 3 cm) were collected from liver segment VI. The liver samples were washed in PBS to remove any blood from the vessels, and then immediately frozen in liquid nitrogen and stored at −80 °C for further analysis. The timeline of the experiment is shown in Figure 1.

### 2.2. Expression of the Genes

The expression of the following selected cytokine genes was measured: interleukin-1α (*IL-1α*), interleukin-1β (*IL-1β*), interleukin-2 (*IL-2*), interleukin-4 (*IL-4*), interleukin-6 (*IL-6*), interleukin-8 (*IL-8*), interleukin-10 (*IL-10*), interleukin-12 (*IL-12*), interleukin-16 (*IL-16*), interleukin-18 (*IL-18*), interferon α (*INF-α*), interferon β (*INF-β*), interferon γ (*INF-γ*), tumor necrosis factor α (*TNF-α*), and chemokine 4 (*CCL4*) in the liver of young, castrated bucks.

The 30 mg frozen liver sample was homogenized (FastPrep^®^-24, MP Biomedicals, Santa Ana, CA, USA) at 10,000 g for 10 min at 4 °C in a buffer containing guanidine thiocyanate and supplemented with protease inhibitor. Total RNA was then isolated using a RNeasy Mini Kit (50) (Qiagen, Hilden, Germany) according to the manufacturer’s protocol. The quantity and quality of RNA was determined using NanoDrop 2000 spectrophotometer (NanoDrop, Wilmington, NC, USA) as well as BioAnalyser 2100 (Agilent Technologies, Massy, France). Only samples with RIN > 7 were used for further analyses. 

Reverse transcription was performed using the Transcriptor First Strand cDNA Synthesis Kit (Roche, Basel, Switzerland). Briefly, the samples of cDNA were diluted to 50 ng/μL on a 96-well plate. RT-qPCR was then performed using the LightCycler^®^ 480 with 2 µL of cDNA samples diluted to 50 ng/μL, 3 µL RNase free water, 0.7 µL of each primer (forward and reverse) and 6.6 µL SYBR^®^ Green I Master (Roche, Basel, Switzerland) on a 96-well plate. The samples were subjected to pre-incubation at 95 °C for 5 min and then run in the following conditions for 45 cycles: denaturation at 95 °C for 5 s, then annealing at 60 °C for 15 s (the same temperature for all genes), and elongation at 72 °C for 20 s. Before amplification, the temperature was established for all studied genes on a temperature gradient using the T100 Thermal Cycler (Bio-Rad Inc., Hercules, CA, USA). All samples were run in triplicate. All procedures were performed according to the manufacturer protocols.

Two reference genes were used in the RT-qPCR procedure: *PPIA* for cyclophilin A and *CLN3* for battenin, as described previously [20]. The gene primer sequences, given in Table 1, were selected based on literature data [21,22,23]. 

The relative gene expression was calculated according to Pfaffl [24], wherein the normalization factor (NF) was obtained as the geometric mean of the crossing point (CP) values of the triplicate readings of the two reference genes. The size of the product was checked on a 2% agarose gel and then visualized under UV light using a G: BOX device (Syngene, Frederick, MD, USA).

### 2.3. Statistical Analysis

The normality of the distribution of the relative mRNA levels was checked using the PROCC UNIVARIATE module (SAS/STAT, 2002–2012, version 9.4) of the SAS package. As the mRNA levels were not normally distributed, they were transposed using the natural logarithm scale for analysis. The transformed data was then subjected to analysis of variance (ANOVA) taking into account group fixed effect (GLM procedure, SAS package; SAS/STAT, 2002–2012, version 9.4). Student’s *t*-test was used as a post hoc test. Pearson’s correlation coefficients were calculated within each group using the PROC CORR module of the SAS package. Relationships were considered significant at *p* ≤ 0.05; however, values between 0.05 and 0.1 were regarded as trends [25,26].

## 3. Results

**Figure 2:**
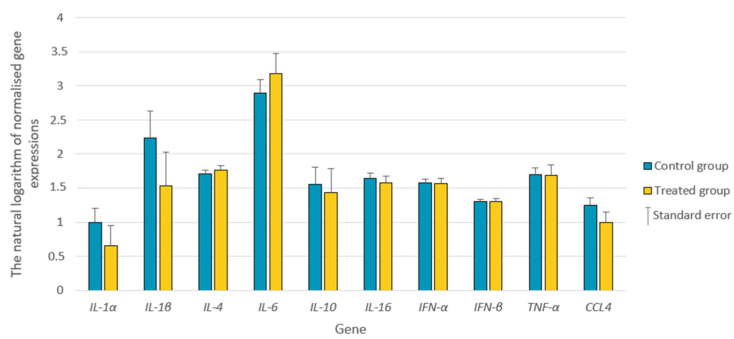
The expression of the studied genes (natural logarithm from normalized gene expressions using two references) in the liver of bucks from the treated and control groups. *IL-1α—*Interleukin-1α; *IL-1β—*Interleukin-1β; *IL-4—*Interleukin 4; *IL-6—*Interleukin 6; *IL-10—*Interleukin 10; *IL-16—*Interleukin 16; *IFN-α—*Interferon α; *IFN-β—*Interferon β; *TNF-α—*Tumor necrosis factor α; *CCL4*—Chemokine 4. For all traits *p* > 0.1.

*IL-2* and *IL-8* expression was below the level of detection in the liver. No differences in *IL-1α*, *IL-1β*, *IL-4*, *IL-6*, *IL-10*, *IL-16*, *IFN-α*, *IFN-β*, *TNF-α*, or *CCL4* gene expression was found between the control and treated groups (Figure 2).

**Figure 3:**
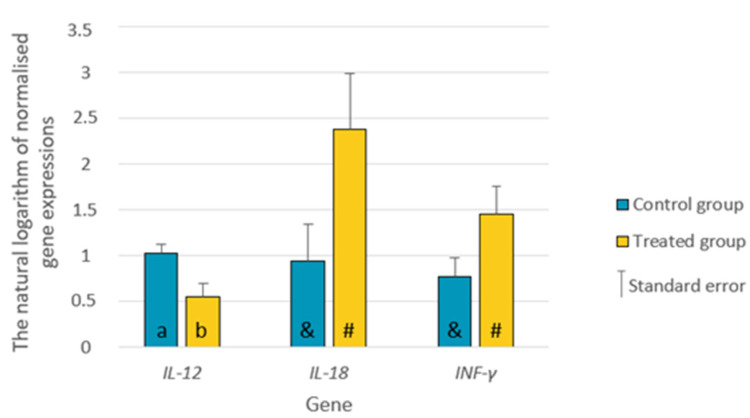
The differences in the relative expression of the selected genes (natural logarithm from normalized gene expressions using two references) between the treated and the control groups. a, b—different letters indicate the significance of differences at *p* ≤ 0.05. &, #—the significance of differences at 0.05 < *p* ≤ 0.10. *IL-12—*Interleukin-12; *IL-18—*Interleukin-18; *IFN-γ*—Interferon *γ*.

However, the expression of *IL-12*, *IL-18* and *INF-γ* differed between the control and treated groups. *IL-12* expression was significantly lower in the treated group (*p* ≤ 0.05), while *IL-18* and *INF-γ* were slightly higher, but only at the trend level (0.05 < *p* < 0.10) (Figure 3).

Only a few correlations were found between the traits demonstrated by the two groups; however, these were quite pronounced. In the control group, significant relationships were found between *CCL4* and *IL-1α* (Pearson’s correlation coefficient: 0.80; *p* < 0.05) and *CCL4* and *IL-1β* (−0.72; *p* < 0.05), *IL-4* and *IFN-α* (0.81; *p* < 0.05), *IL-4* and *IFN-β* (0.85; *p* < 0.05), *Il-16* and *IL-18* (−0.92; *p* < 0.01), and *IFN-α* and *IFN-β* (0.94; *p* < 0.01). In the treated group, no correlations were found between *CCL4* and any other genes. However, *IL-4* was positively correlated with *IFN-α* (0.98; *p* < 0.01), *IFN-β* (0.99; *p* < 0.01), and *IL-16* (0.98; *p* < 0.05). *IFN-β* was also positively correlated with *Il-18* (0.98; *p* < 0.05). In addition, a negative correlation was found between *IL-12* and *IL-1β* (−0.97; *p* < 0.05) as well as between *IL-12* and *IL-18* (−0.93; *p* < 0.05). No correlation was found between *IFN-γ* and any other trait in either group.

## 4. Discussion

Although the Polish goat market represents only a small part of the agricultural economy, the demand for goat products has been rising for a long time, with the dairy product market currently growing by 10–15% per year [27]. However, while goat meat is regarded as a functional food in many countries [28], it only has a small share of the market in Poland [27]. To promote goat meat and develop its market, it is essential that goat meat has an appropriate composition and comes from healthy animals [1]. As polyphenols and curcumin in a proper doses are known to have beneficial effects on human and animal health, we hypothesized that a turmeric-rosemary dry mixture would influence cytokine expression as part of the immune system of young, castrated bucks. The roles of cytokines and their division into pro- or anti-inflammatory activities are presented in Table 2. Some of them are classified as pleiotropic cytokines because they act differently on different types of cells.

IL-2, IL-4 and IL-10 participate in the immune system by stimulating T and B cell activities. Studies on human, mouse, and rat tissue have found curcumin to inhibit the expression of pro-inflammatory cytokines, including *IL-2* and *IL-4*, in several tissues or organs, including the liver [39]. The main role of IL-2 is to activate the growth, proliferation, and differentiation of progenitor and mature cells. It participates in the development of Treg cells (regulatory lymphocytes T), thus inhibiting the immune response and protecting against autoaggression [40]. However, no *IL-2* expression was found in the studied buck liver tissue in either group in the present study. This difference may be due to our decision to use a spice-herb mixture. While the content of the rosemary extract was low in the mixture, it is possible that the polyphenol constituents of one spice might influence the others; however, this requires further study. As mentioned above, *IL-2* was not expressed in either group. Instead, *IL-4* and *IL-10* transcripts were observed in liver tissue, where they would have had anti-inflammatory and anti-autoaggression activities. However, supplementation did not appear to influence their production. While IL-2 is the most important growth factor for T lymphocytes and NK cells, IL-4 and IL-10 are anti-inflammatory cytokines that inhibit pro-inflammatory cytokines. Therefore, the fact that the tested mixture had no apparent effect on their expression suggests that the supplement did not inhibit inflammatory reactions. 

Moreover, Guo et al. [41] found elevated *IL-10* gene expression in the hepatocytes of goats treated with the polyphenol quercetin; however, this substance is absent in the turmeric-rosemary mixture. No such elevated expression was observed in the present study, which suggests that none of the other polyphenols present in the studied mixture influenced *Il-10* expression, or their level was too low to affect the transcription processes. 

Similarly to IL-16 and TNF-α, IL-8 also demonstrates chemokine activity. *IL-8* expression is also inhibited by various supplements, including curcumin, which has been found to inhibit *TNF-α* in bleeding rats and block *TNF-α* production in LPS-stimulated macrophages; hence, the anti-inflammatory activity of curcumin may be associated with its ability to inhibit the activity of inflammatory cytokines [39]. A study found that goats fed with a high concentrate and selenium-supplemented diet (HCSe) or a low-concentrate diet (LC) demonstrated lowered liver *TNF-α* expression than those fed a high concentrate diet alone (HC); however, no difference was found between HCSe and LC goats [42]. Finally, IL-16 inhibits T cell proliferation, promotes Th1-dependent responses, and reduces Th2-mediated inflammation by activating the release of TNF-α and IL-1β, while also inhibiting the production of IL-4 [43]. Previous studies have found feeding an excessively concentrated and high-grain diet to increase the concentration of pro-inflammatory cytokines (IL1-α, IL1-β, IL-6, TNF-α, IL-8) in the liver and to induce an inflammatory response [44,45]. However, supplementation did not appear to affect the expression of these groups of genes in liver cells in the present study, suggesting that homeostasis was maintained.

IL-12 plays an important role in the immune response during bacterial, protozoa, and fungi infections by activating inflammatory cells (NK cells, monocytes, macrophages, neutrophils microglia, and dendritic cells), generating lymphokine-activated killer cells (LAKs) and inducing T cell proliferation and IFN-γ production [46]. IL-18 is produced by various immune cells and non-immune cells, such as intestinal epithelial cells. However, the functions of IL-12 and IL-18 differ according to cell type [43]. While IL-18, in collaboration with IL-12, stimulates Th1-mediated immune responses that play a key role in the host defense through the induction of IFN-γ, overproduction of IL-12 and IL-18 induces severe inflammatory disorders. This suggests that IL-12 and IL-18 are potent pro-inflammatory cytokines that can have a pathophysiological role during inflammation [47,48]. The stimulatory ability of IL-18 regarding leukocytes allows it to participate in various allergic responses, including rhinitis, dermatitis, asthma, and eosinophilic disorders. In addition, IL-18 can stimulate mast cells, basophils and other granulocytes from bone marrow precursors; interestingly, while it can also induce B cell expansion and isotype switching, IL-12 inhibits these [49]. Thus, the increased expression of *IL-18* indicates an allergic or sensitized reaction [47], i.e., a hypersensitive state involving various immune mechanisms mediated by IgE and leukocytes. In the present study, the turmeric-curcumin mixture was found to inhibit *IL-12* expression, which is consistent with Jagetia and Aggarwal [39] results. Therefore, the presence of lowered *IL-12* expression together with elevated *IL-18* mRNA expression may suggest that the supplementation may have elicited an allergic or sensitized reaction in our goats. Indeed, a strong negative correlation was found between *IL-12* and *IL-18* gene expression. Therefore, it is possible that the herb-spice mixture dose, originally recommended for dairy goats, was too high for the smaller bucks in the study, which were not metabolically burdened by milk production. This is especially important considering that curcumin is the main component of the mixture.

It is possible that the elevated *IFN-γ* expression observed in the treated group may have been caused by increased expression of IL-18, despite *IL-12* gene expression being reduced. However, no correlation was observed between *IFN-γ* and any other genes. Interferons (IFNs) are produced by macrophages, fibroblasts, endothelial cells, and specialized leukocytes, known as interferon-producing cells. Type I IFNs such as IFN-alpha (IFN-α) and IFN-beta (IFN-β) exhibit antiviral activity by inhibiting viral replication, increasing the natural killer (NK) cell lysis potential, influencing the expression of MHC class I molecules in virus-infected cells and stimulating Th1 cell growth. Type II IFNs, of which the only known example is IFN-γ, play a major role in the activation of macrophages in both the innate and adaptive immune responses [43,50,51]. IFN-γ is secreted by activated T cells and natural killer (NK) cells. IFN-γ induces hepatocyte apoptosis and inhibits cell cycle progression during liver diseases. In the present study, *IFN-α* and *IFN-β* transcripts were found to be present at similar levels in the control and treated groups, which could indicate a lack of any viral infection. However, increased INF-γ expression, known to activate macrophages, may indicate a high readiness of the non-specific immune system to react quickly in case of pathogen invasion. However, the higher level of *IFN-γ* transcripts in the treated group may also indicate the presence of triggered lymphocyte T and NK cells, which could help overcome autoimmunity or inflammation [40]. 

In summary, supplementation with the herb-spice mixture containing polyphenols did not appear to result in any changes in the expression in most studied genes, particularly pro-inflammatory cytokines; this might suggest that the mixture did not have a negative or positive influence on the homeostasis of the healthy bucks. It also appears that based on the test described by McKenna et al. [52], the diet of the castrated bucks was balanced with regards to energy. However, increased expression of *IL-18* and *INF-γ* was noted, together with decreased expression of *IL-12*, which may indicate some form of hypersensitivity; this could be due to the dosage of the supplement being too high. Unfortunately, allergen hypersensitivity can be subclinical and a firm diagnosis also requires a positive test for the presence of a specific serum allergen IgE (sIgE) [53]. As the dose of the supplement was established by the producer based on the previous experiences with dairy cow nutrition, it was not anticipated that this dose could be too high for the bucks in the study and *IgE* gene expression was not included in the study. 

Future studies could examine the influence of different doses of the mixture on immune gene expression and include various biochemical parameters and/or oxidative stress biomarkers in the blood serum of treated animals, especially ALT and AST, i.e., indicators of liver function, as well as mast cell activity. It should be stressed that hypersensitivity could gradually result in the onset of allergic disease [52] and establishing the proper dose of the supplement is essential.

## 5. Conclusions

Supplementation with the *C. longa* and *R. officinalis* extract mixture did not appear to result in any significant change in the levels of most of the studied pro-inflammatory cytokines. This may mean that supplementation does not activate hepatocytes for cytokine production in healthy goats. However, treatment resulted in reduced *IL-12* gene expression and slightly higher *IL-18* gene transcript levels, which may indicate a hypersensitivity reaction caused by an excessive dose of the supplement. In addition, increases in pro-inflammatory *IFN-γ* transcript levels were noted, which may also indicate that the supplement has pro-inflammatory effects.

These findings, that a small dose of extract mixture can induce an allergic reaction in 30–40 kg goat bucks, suggest that high levels of curcumin and/or turmeric extract may also be harmful to humans.

## Figures and Tables

**Table 1:** Gene name, symbol, primer sequences, amplification product sizes, GenBank accession number and references for primer sequences.

Group of Genes	Gene Name	Gene Symbol	Primer Sequence		Product Size (bp *)	GenBank ^/UniProt Accession	Reference
References	Cyclophilin A	*PPIA*	F	GGATTTATGTGCCAGGGTGGTGA	120	AY_247029.1	[22]
			R	CAAGATGCCAGGACCTGTATG			
	Battenin	*CLN3*	F	TTCTGACTCCTTGGGACACA	62	NM_001075174	[22]
			R	CAACCTGCCCACCTATCAGT			
Cytokines	Interleukin-1α	*IL-1* *α*	F	TGAACGACGCCCTCAATCAA	366	D63350.1	[23]
			R	CTTGCCATGTGCACCAGTTT			
	Interleukin-1β	*IL-1* *β*	F	GCAGCTGGAGGAAGTAGACC	231	D63351.1	[23]
			R	TGGCTTTCTTTAGGGAGAGAGG			
	Interleukin-2	*IL-2*	F	ATGTACCAGATACCACTCTTGTCTT	467	AY603404.1	[23]
			R	TCAAGTCATTGTTGAGTAGAT			
	Interleukin-4	*IL-4*	F	TAGCTTCTCCTGATAAACTA	534	U34273.1	[23]
			R	ATGAGTTATAAATATATAAATA			
	Interleukin-6	*IL-6*	F	TCTTCACAAGCGCCTTCAGT	120	D86569.1	[23]
			R	CTGCTTGGGGTGGTGTCATT			
	Interleukin-8	*IL-8*	F	TGAGAG TGGGCCACACTGC	103	JN559767.1	[21]
			R	CACAACCTTCTGCACCCACTT			
	Interleukin-10	*IL-10*	F	CGGCGCTGTCATCGTTTT	82	DQ837159.1	[23]
			R	TCTTGGAGCATATTGAAGACTCTCTTC			
	Interleukin-12	*IL-12*	F	CACCAAAGATAAAACCAGCACAGT	125	AY603407.1	[23]
			R	GTCTTTCCAGAAGCCAGACAATG			
	Interleukin-16	*IL-16*	F	AAAAGACCTCTGCGGGACTG	213	AF481158.1	[23]
			R	TCAGGCAACGCCTTGATGAT			
	Interleukin-18	*IL-18*	F	TCCTAAGAAGCTATTGAGCACAGGC	619	AY605263.1	[23]
			R	ATTTTAATATCTAGTCTGGTTTTG			
	Interferon α	*IFN-α*	F	CACCTTCCAGCTCTTCAGCA	96	FJ959074.1	[23]
			R	GTCAGTGAGCTGCTGATCCA			
	Interferon β	*IFN-β*	F	ACAGCAGTTCCGGAAGGAAG	212	JX458085.1	[23]
			R	TCGGTCGTGTCTCCCATAGT			
	Interferon γ	*IFN-γ*	F	TAGCTAAGGGTGGGCCTCTTTTCTCA	384	AY603405.1	[23]
			R	TGCAGGCAGGAGAACCATTACATTGA			
	Tumor necrosis factor α	*TNF-α*	F	AGAAGGGAGATCGCCTCAGT	171	X14828.1	[23]
			R	AGAAGGGGATGAGGAGGGTC			
	Chemokine 4	*CCL4*	F	CAGCCGTGGTATTCCAGACC	109	XM_005693171.3	[21]
			R	CTCGGAGCAGCTCAGTTCAGT			

^ NCBI (http://www.ncbi.nlm.nih.gov, accessed on 15 October 2015); * bp—base pairs; F—forward primer; R—reverse primer.

**Table 2:** A list of the pro- or anti-inflammatory cytokines examined in the present study.

Gene Name	Gene Symbol	Immune Activity	References
Interleukin-1α	*IL-1* *α*	Proinflammatory	[29]
Interleukin-1β	*IL-1* *β*	Proinflammatory	[30]
Interleukin-2	*IL-2*	Pro- and anti-inflammatory	[31]
Interleukin-4	*IL-4*	Pro- and anti-inflammatory	[32]
Interleukin-6	*IL-6*	Proinflammatory	[30]
Interleukin-8	*IL-8*	Proinflammatory	[33]
Interleukin-10	*IL-10*	Anti-inflammatory	[30,34,35]
Interleukin-12	*IL-12*	Proinflammatory	[36]
Interleukin-16	*IL-16*	Pro- and anti-inflammatory	[37]
Interleukin-18	*IL-18*	Proinflammatory	[30]
Interferon α	*IFN-α*	Proinflammatory	[34]
Interferon β	*IFN-β*	Proinflammatory	[34]
Interferon γ	*IFN-γ*	Proinflammatory	[38]
Tumor necrosis factor α	*TNF-α*	Proinflammatory	[30]
Chemokine 4	*CCL4*	Proinflammatory	[38]

## Data Availability

The datasets used and/or analyzed during the current study are available from the corresponding author on reasonable request.

## Abbreviations

CCL4, chemokine 4; CLN3, battenin; IL-10, interleukin-10; IL-12, interleukin-12; IL-16, interleukin-16; IL-18, interleukin-18; IL-1α, interleukin-1α; IL-1β, interleukin-1β; IL-2, interleukin-2; IL-4, interleukin-4; IL-6, interleukin-6; IL-8, interleukin-8; INF-α, interferon α; INF-β, interferon β; INF-γ, interferon γ; PPIA, cyclophilin A; PWI, Polish White Improved; SRLV, small ruminant lentivirus; TNF-α, tumor necrosis factor α.
